# Biocoagulation of Dried Algae *Chlorella* sp. and Pellets of *Aspergillus Niger* in Decontamination Process of Wastewater, as a Presumed Source of Biofuel

**DOI:** 10.3390/jof8121282

**Published:** 2022-12-07

**Authors:** Alžbeta Takáčová, Miriama Bajuszová, Alexandra Šimonovičová, Štefan Šutý, Sanja Nosalj

**Affiliations:** 1 Department of Environmental Ecology and Landscape Management, Faculty of Natural Science, Comenius University, Ilkovičova 6, 84215 Bratislava, Slovakia; 2 Department of Soil Science, Faculty of Natural Science, Comenius University, Ilkovičova 6, 84215 Bratislava, Slovakia; 3 Department of Wood, Pulp and Paper, Faculty of Chemical and Food Technology STU, Vazovová 5, 81243 Bratislava, Slovakia

**Keywords:** *Aspergillus niger*, *Chlorella* sp., decontamination, adhesion

## Abstract

The removal of microalgae represents a problematic part of the water decontamination process, in which most techniques are expensive and non-ecological. In the paper, we focus on the synergistic relationship between microscopic filamentous fungi and algal culture. In the process of decontamination of a model sample containing ammonium ions, efficient biocoagulation, resp. co-pelletization of dried algae *Chlorella* sp. and *Aspergillus niger* sensu stricto are shown. The microscopic filamentous fungus species *A. niger* was added to a culture of an algal suspension of *Chlorella* sp., where the adhesion of the algal cells to the fungi subsequently occurred due to the electrostatic effect of the interaction, while the flocculation activity was approximately 70 to 80%. The algal cells adhered to the surface of the *A. niger* pellets, making them easily removable from the solution. The ability of filamentous fungi to capture organisms represents a great potential for the biological isolation of microalgae (biocoagulation) from production solutions because microalgae are considered to be a promising renewable source of oil and fermentables for bioenergy. This form of algae removal, or its harvesting, also represents a great low-cost method for collecting algae not only as a way of removing unnecessary material but also for the purpose of producing biofuels. Algae are a robust bioabsorbent for absorbing lipids from the environment, which after treatment can be used as a component of biodiesel. Chemical analyses also presented potential ecological innovation in the area of biofuel production. Energy-efficient and eco-friendly harvesting techniques are crucial to improving the economic viability of algal biofuel production.

## 1. Introduction

Microalgae are ideal for alleviating the current situation with the depletion of fossil carbon sources and the increasing concentrations of CO_2_ in the atmosphere. The ability of algae to absorb and adsorb metals was recognized many years ago. Algae are the main regulator of heavy metals in aquatic environments due to their ability to take up toxic substances from the environment, and bioaccumulation studies point to the intake of contaminants via different paths [1,2]. One of the main reasons why the production of microorganisms is considered promising is the possibility of using several aspects of the harvested product. Oil is considered to be the most promising volume product from microalgae, as it can achieve a yield of 80% of the dry weight of some types of algae and then be processed into biodiesel based on already developed methods [3].

At present, the use of algae for the preparation of biodiesel is being shown as possible. Microalgae, in view of their multi-purpose effects on water and air, are being studied as a sustainable source for the production of biodiesel. The extraction of oil and its direct transesterification can potentially establish microalgae as a next-generation fuel source [4]. For economically feasible use of microalgae as a basis for bioenergy, the costs of the nutrient and carbon sources should be minimized, which can be conducted by utilizing the output streams from other processes. An example is wastewater from municipal and agricultural sources, which is rich in nitrogen and phosphorus content, and from industrial production enriched with CO_2_. Facilities for the cultivation of algae can be used as appropriate inlet flows [5]. Algal biomass, after lipid extraction and biofuel production, has the potential for biosorption and/or the neutralization of the toxic effects of heavy metal ions from industrial wastewater [6]. The use of microalgal feedstock as a third-generation biofuel has gained significance due to sustainability and feasibility. Compared to terrestrial plants, it has a higher growth rate and superior CO_2_ and sunlight fixation capacity along with higher lipid content. Energy-efficient and eco-friendly harvesting techniques are crucial to improving the economic viability of algal biofuel production [7].

The main disadvantage of autotrophic cultures compared with heterotrophic cultures is the low cell density. Despite the optimization of cultivation conditions, the isolation of microscopic and easily dispersed algae from the aquatic environment is still a barrier to commercial processing [8]. It is assumed that microalgae can be a universal solution for converting contaminants, and enabling the recycling, reuse and safe release of treated water. The technology is also low-cost and represents an effective approach to removing excess nutrients and contaminants from wastewater and generating potentially useful biomass [9,10,11]. The benefit of algal microorganisms lies in their capacity for rapid growth, the high productivity of lipids (palmitic acid (C16:0), linoleic acid (C18:2) and y-linoleic acid (C18:3)), potential in bioremediation technology and various valuable by-products. The harvesting of microalgae is considered one of the key points in biofuel production, representing minimally 20–30% of the total production costs and limiting production on a commercial and industrial scale [12].

Collection and drainage equipment can represent up to 90% of total costs in the case of microalgae production in open ponds [13,14]. Harvesting the biomass of microscopic algae from a cultivation solution is one of the main challenges that has limited the widespread use of algae as raw material for biofuels. There are many technologies for harvesting microalgae, but the majority of them are either labor-intensive, energy-intensive or environmentally unfriendly [15,16,17,18].

*Aspergillus niger* is a type of microscopic filamentous fungus with a cosmopolitan distribution in all components of the environment [19]. It is a major producer of several organic acids, metabolites, as well as mycotoxins and enzymes, such as lipase [20,21]. *A. niger* has uses in various branches of industry, particularly in the pharmaceutical and food industries. The ability of this species to absorb many harmful components from the environment, including heavy metals and toxic metals, also has uses in the decontamination of various substrates. In the industrial application of microscopic filamentous fungi, their biomass is used in the form of pellets [22,23]. In the process of bioflocculation, this species is also very actively used in the harvesting of algae and for bulk biomass production [24,25,26].

The main aim of this study was to investigate the synergism of the microscopic filamentous fungus *A. niger* and an attenuated biomass of the algal culture *Chlorella* sp. in the process of decontaminating ammoniacal waters; finding this synergism to also be useful for algae biocoagulation is certainly pleasing. Monitoring of the algae and its synergism for use in the process of creating biofuel is also appropriate. One of the most environmentally friendly and sustainable methods for biofuel sources is using microalgae for the production of lipids and wastewater as its nutrient source [27]. The accumulation of lipids by algae and their subsequent flocculation with the help of pellets represents the industrial potential for the production of biodiesel. As was shown by Kamyab et al. (2014) [28] optimal cultivation of microalgae growth causes biomass growth and leads to higher lipid production. Thanks to the subsequent thermochemical conversion, the use of microalgal and fungal synergism means the elimination of a large number of previously used processes, which is expected to rapidly reduce production costs.

## 2. Materials and Methods

### 2.1. Selection of Species for the Experiment

Two different microorganisms were used in the studies, the autotrophic algae *Chlorella* sp. and the fungus *A. niger* as a heterotrophic representative of microscopic filamentous fungi. The strain *A. niger* sensu stricto marked An-Š was isolated from Dystric Cambisol (contaminated and eroded) without vegetation in Banská Štiavnica-Šobov from a depth of 5–10 (15) cm. The soil substrate is ultra acidic (pH 3.12) with an exceeded value of aluminum (727–506 mg/kg) and very poor on organic material (% C_ox_ 0.49). Isolates of strain *A. niger* were identified in previous work [29]. The dilution plate method (dilution 104 CFU) was used for the isolation of cultivable microscopic fungi. Mixed cultures were cultivated in the dark at a temperature of 25 °C. The strain *A. niger* was purified by several cultivation steps on Sabouraud Dextrose Agar (SAB), Czapek Dox Agar (CD) and Czapek Dox Agar w/Yeast Extract (CDY) agars. Pure cultures were identified according to their micromorphological features using the diagnostic keys [30,31] and according to their Internal Transcribed Spacer (ITS) sequences. The resulting sequence was directly compared with those in GenBank using BLAST searches (accessed on 19 March 2021, https://www.ncbi.nlm.nih.gov/nuccore/MW739953). The selection of the mentioned strain was targeted and focused on its behavior in different model solutions. We also assumed its high adaptability to environmental changes. In the case of microalgae, the dry biomass of the algae culture *Chlorella* sp. was obtained from the Institute of Microbiology of the Academy of Sciences of the Czech Republic, Opatovický Mlýn, was used.

### 2.2. Parameters of Used Waters

Three types of water were used in this experiment. Distilled water, model water and real wastewater. Wastewater was collected from the mechanical stage of a wastewater treatment plant (WWTP). The characterization of the water is shown in Table 1.

### 2.3. Preparation of A. Niger Pellets

*A. niger* fungal pellets were prepared in 45 mL of SDB (Sabouraud Dextrose Broth Liquid Medium, Himedia, Mumbai, India) enriched with a 5 mL suspension of conidia from a pure culture of *A. niger*. Cultivation took place over 5 days under laboratory conditions at 25 °C in 250 mL Erlenmeyer flasks, with stirring at 170 rpm (Unimax 2010 shaker, Heidolph, Germany). The fungal pellets formed very quickly, and after 5 days they were removed by filtration, washed with a large amount of distilled water and used in the experiments [22].

### 2.4. Biocoagulation of the Algae

Pellets of *A. niger* were added to a suspension solution of *Chlorella* sp. in distilled water with a concentration of algal biomass of 5 g.L^−^^1^ in a total volume of 50 mL. Sterile 100 mL Erlenmeyer flasks in three repetitions were placed on a shaker for 24 and 48 h at 150 revolutions/min. The experiment took place at a laboratory temperature of 25 °C under natural light for 8/16 h. A monoculture of the fungi and green algae was prepared under the same conditions and used as the control samples.

### 2.5. Chemical Analysis of Elements

The content of selected biogenic elements (N, C, H and S) was determined by means of an Elementar Vario Macro Cube (Germany). The principle of the method consists in burning the sample at a high temperature of up to 1200 °C in the presence of oxygen, and the injection of oxygen leads directly into the sample, thus ensuring the highest concentration of oxygen at the point of combustion and low gas consumption.

### 2.6. Analysis of the Surface Charge of the Dispersed Particles

The surface charge of the dispersed particles was measured using the CAS touch Charge Analyzing System (AFG ANALYTIC GMBH, Germany). Cationic PDADMAC (poly-diallyl-dimethyl-ammonium chloride) and anionic PVS Na (polyvinyl sulfonic acid sodium salt) were used as standard polyelectrolytes, both with a concentration of 0.001 n [32].

### 2.7. Microscopy

The photodocumentation of *A. niger* pellets and dry green algae biomass was made with a Canon IXUS 16.1-megapixel camera (Japan). Details of the *Chlorella* sp. surface structure were observed under a JEOL JXA 840A Scan Electron Microscopy (SEM) (Japan). Microscopic observation of the *A. niger* and *Chlorella* sp., as well as *A. niger* with *Chlorella* sp. pellets, were made with an Axio Scope A 1 Carl Zeiss Jena light microscope in a drop of lactic acid enriched with a cotton blue stain (0.01%).

## 3. Results

In our study, 5 g/l of the algal culture was weighed into 50 mL Erlenmeyer flasks, to which prepared pellets of *A. niger* of size 5–7 mm were added. The mixture was filled up to a total volume of 50 mL with distilled water in three parallels. The same procedure was followed in the case of the model as well as real wastewater from the mechanical stage of a wastewater treatment plant (WWTP) (Table 1).

**Figure 1:**
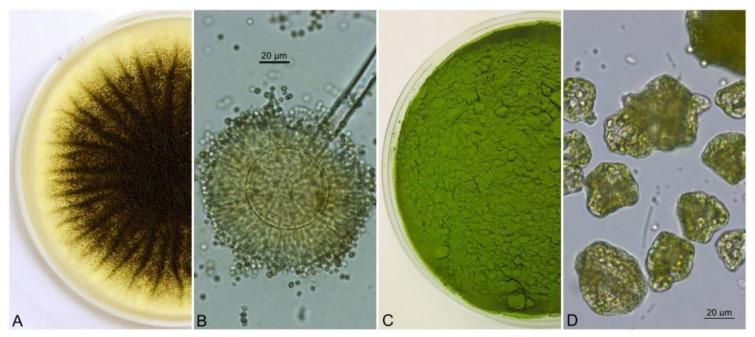
Cultivation of *A. niger* strain on SDA (Sabouraud Dextrose Agar) (**A**), conidial head with conidiophore terminated by a vesicle with phialides and conidia (**B**), dried culture of the green alga *Chlorella* sp. (**C**), dried culture of *Chlorella* sp. under a light microscope (**D**).

Pelletization experiments were conducted without pH adjustment and without targeted ionization. The source of nutrients for *A. niger* was the *Chlorella* sp. algal culture. The applicability of coagulation is determined by the aggregate stability of the removed contaminant in water and the factors that condition the colloidal nature (temperature, pH, zeta potential, conductivity) [33]. The experiment was repeated by adding already pre-grown pellets of *A. niger* in a volume of 5 mL to the algal suspension. Figure 1A–D presents different structures of model organisms used in this study.

The flocculation activity was calculated from Equation (1).

Flocculation activity (%) = (A − B) A × 100 (1)

where A = Absorbance of the algal culture at 680 nm at the start in the control, B = Absorbance at 680 nm of the algal culture after pelletization.

The pellets showed high flocculation efficiency (flocculation activity in the range of 70–80%).

**Figure 2:**
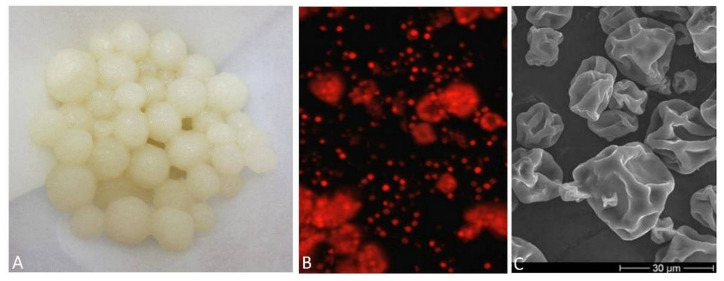
*A. niger* pellets after washing in distilled water (**A**), dry culture of the algae *Chlorella* sp. under a fluorescence microscope (**B**), record of a dry culture of the algae *Chlorella* sp. from a scanning electron microscope (**C**).

The principle of measurement is based on the electrokinetic potential of moving dispersed particles using the streaming current detector (SCD) current-voltage method and with charge compensation by polyelectrolyte. The instrument generates motion in the sample, which enables the movement of the mobile counterion layer (which surrounds the particle) [32,34,35]. During the experiment, the initial charges of the pellets and algae were also measured; the algae appeared in the environment as negatively charged, while the pellets of *A. niger* (Figure 2A), on the other hand, had a positive initial charge.

The specific charge density was calculated based on Equation (2).

Specific charge density (meq/g) = f_c_ nVw ^−1^ (2)

Where f_c_ = 1000 is the conversion factor for the unit charge density, *n*—is the concentration of the standard polyelectrolyte (mol/L), V—is the volume of the polyelectrolyte titer (mL), w—is the content of dispersed or dissolved material in 10 mL of sample solution (mg) or (mL) [32,34,35]. The results of the specific charge density measurements are presented in the Table 2.

The charge of algae particles does not change with the time of biocoagulation. The charge depends on the different components of the solution. The amount of negative surface charge of algae particles depends on the composition of the solution. This is consistent with the theory that the ionic strength of the solution affects the surface charge. As shown in Table 2., for algae solutions with a concentration of 0.5 g/L, the size of the negative charge increases in the following order: distilled water—solution of ammonium ions 9 mg/L + 0.5 g/L algae biomass—solution of ammonium ions 9 mg/L + 0.5 g/L algae biomass + 5 g/L pellets, while for algae solutions with a concentration of 5 g/L, the size of the negative charge increases in the following order: distilled water—solution of ammonium ions 9 mg/L+ 0.5 g/L algae biomass + 5 g/L pellets—solution of ammonium ions 9 mg/L+ 0.5 g/L algal biomass.

At the end of the experiment (after 72 h), the content of the selected biogenic elements (C, N, H, and S) in the biomass (algae and pellets) was determined using the Elementar Vario Macro Cube (Germany) (Table 3).

The concentration of non-metals in the resulting biomass after the process of coagulation in the model sample is shown in Table 3. The highest contribution after 72 h of coagulation was approx. 46% for the carbon content in the case of a concentration of 5 g/L of *Chlorella* sp. together with *A. niger* pellets. The concentration content of non-metals (C, H, N, S) in the biomass is important for subsequent evaluation; for example, in biogas stations for the production of biomethane, particularly in this time of energy shortage. Biogas stations wrestle with a lack of plant biomass (reduced agricultural production) as well as the ban on using wood biomass in biogas stations.

In our experiment, unlike in other studies, a dried algal culture (partially freeze-dried) was used. The presence of intact algal cells was recorded by a scanning microscope as documented in our previous experiments (Figure 2C) [22,36]. The destruction of the cell wall in the algal biomass used for pelletization was not determined. The membrane remains intact, which means that enzyme activity in the reaction center is possible.

The presence of chlorophyll is also evidenced by the output from the fluorescence microscope, where the red coloring corresponds to the presence of chlorophyll (Figure 2B).

**Figure 3:**
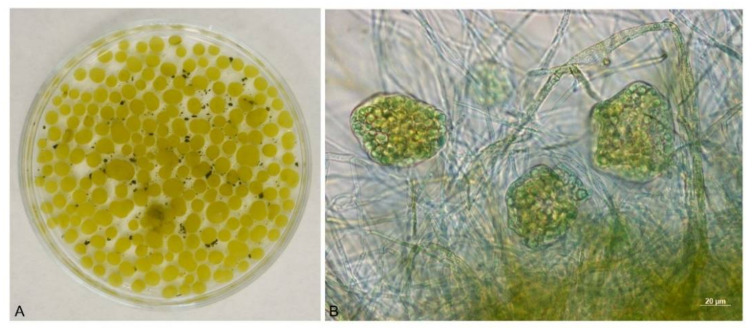
*A*. *niger* pellets with *Chlorella* sp. after cultivation on a shaker (**A**), detail of the “hairy region” of the *A. niger* pellet with *Chlorella* sp. (**B**).

The fluorescence of living systems can be used for the determination of the potential damage to the photosystem. In these methods, chlorophyll (CHL) represents an internal probe of an organism’s photosynthetic capacity. For chlorophyll (a), two maxima are crucial: one in the red region near 690 nm and another in the far-red region, near 735 nm [37]. As shown in Figure 3A,B, the biocoagulation of algae with the use of *A. niger* pellets had a relatively high efficiency, as only a very small percentage of non-coagulated algae remained in the solution.

The interaction of pellets and algae formed a complex, which can be directly observed under a light microscope. A large number of fibers on the pellets were interwoven together, resulting in the formation of a compact material visualized as a network structure.

**Figure 4:**
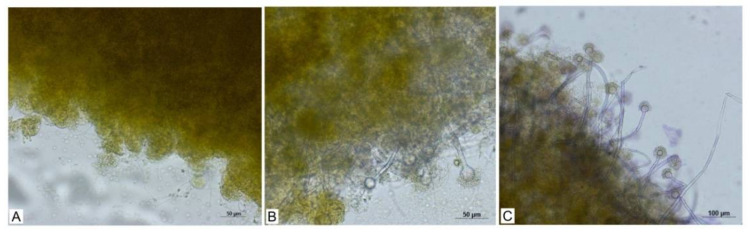
Visual results of biocoagulation. Pellets of *A. niger* (**A**), cells of the algae *Chlorella* sp. (**B**), results after 24 h (**C**), results after 48 h.

The combination of the pellets of *A. niger* with the attenuated biomass of the algal culture *Chlorella* sp. occurred despite the fact that the algal cell was not living. In a timeframe of 24 h, an aggregation of the algal cells was also observed. The formation of microorganisms complex (microscopic filamentous fungus and algal biomass) is possible even with a relatively high concentration of algal suspension in the solution (5 g.L^−1^); 48 h after the start of the experiment, more than 80% pelletization occurred without any changes in the conditions. The change in the state of the pellets at the start and after time dispersions of 24, 48 and 72 h in the algal suspension is documented in Figure 4A–C.

## 4. Discussion

The main process of biocoagulation is adhesion—a process in which a biological or other particle is attached to a surface separately from the particle itself [38]. The adhesion of algae to the fungal cell wall has been the subject of long-term research. As early as 1972 the authors Guggenheim and Haller described an alpha-1,3-glucan-degrading enzyme from the species *Trichoderma harzianum*. Based on this study this polysaccharide was used as a significant sign of a microalgae adhesion ability and studied in *A. niger* species [39]. When searching for homologs of the gene Ags ANI_1_1472184, the encoding of α-1,3-glucan synthase was found in 96 species of fungi, but not in *A. niger* and *T. harzianum*. A comparison of strains from previous studies on the fungal pelletization of microalgae indicates that the existence of an Ags-homologue is not required for fungal pelletization. The missing homolog in strains belonging to *Aspergillus* species indicates different mechanisms, within the same genus, for pellet formation. The weak co-pelletization with *Aspergillus oryzae* shows that the existence of -1,3-glucan in the cell wall is no guarantee of good adhesion of microscopic algae [40].

*A. niger* has a specific microscopic morphology that provides a characteristic way of pellet formation, where an immediate aggregation of conidia occurs after inoculation, representing the first stage of pelletization. During this stage, the pH is increasing. Subsequently, aggregation takes place, initiating the process of germination and hyphal growth of the germ tubes, which increase their surface area and enable conidia to attach, resulting in the formation of pellets. The structure of fungal pellets depends on several factors, including genetics, cell wall composition, inoculum size, growth rate, nutrition, pH, etc. [41,42,43,44,45,46].

Flocculation represents the aggregation of unstable and small particles through surface charge, neutralization, and electrostatic patching—which allows separation by simple gravitational settling—or any other conventional method. The flocculation process takes place gradually; in the first phase, the negative surface charge of the cell is interrupted, by which polymeric or colloidal absorption begins. In the second phase, the interaction of surface-neutral microalgae cells occurs on the basis of the thermodynamic balance of interaction energies, and the third phase represents the local attachment of charged polymers or colloids to the microalgae surface. In the fourth phase, the charged polymers or colloids form bridges between two cells, and the last phase involves the massive entrapment of cells into a precipitate [14]. The flocculation process is mostly simple and efficient and is a promising strategy for harvesting different types of algae [47,48,49,50]. Flocculation by the action of microscopic filamentous fungi and its mechanism has been observed in connection with microalgae [51].

The flocculation of microorganisms supported by fungi shows considerable potential for resolving the main challenges, as many filamentous fungi are known for having self-pelletizing capabilities through which algal cells can be trapped and adsorbed [52]. The addition of calcium can at the same time also bind the cell surfaces of the pellets and algae themselves. Flocculation is a complex process influenced by cell-surface properties, cell concentration, as well as environmental pH, ionic strength, and the type and dosage of the flocculant [53].

A part of the evaluation was also the measuring of the surface tension of the dispersed particles. Even though most biomass in the environment appears to be negatively charged, some microorganisms are able to appear positively charged. It is assumed that the positive surface charge of the biomass of microscopic filamentous fungi is what causes the algae to bind and anchor to their surface. At the start of the measurements, the algae showed a negative charge, while during measurement, the microscopic filamentous fungi had a positive charge. Similar results were observed in the study by Lal et al. [7], which states that during bioflocculation, significant changes occur in the surface groups of the algal and fungal pellets. In the course of bioflocculation, neutralization of the surface charges of the algae and fungi occurs, and the algae are subsequently anchored between the hyphae of the pellets. Therefore, it is correct to say that this change of charges is responsible for the collection of microalgae and represents the potential of this method for biofuel production.

The great advantage is that the collected biomass of microscopic filamentous fungi and algae can be successfully used as a starting material for the production of various biofuels, such as biodiesel, alcohols and biogas, by fermentation and thermochemical methods [54]. Lipids contained in algae represent a potential source for the production of biofuels, either through subsequent biochemical transformation or thermal conversion. Methods of thermochemical conversion include gasification, pyrolysis, hydrogenation and liquefaction of the algal biomass to obtain gas or oil biofuels as well as hydrogen that can be produced from algae by biophotolysis [55,56,57,58].

The growth of microalgae runs in three modes, which affect their growth as well as the content of individual substances in the cells. The production of microalgal biomass as well as the lipid content of microalgal cells in a heterotrophic environment has been observed as being much higher than during phototrophic growth [46,59,60]. As Xia et al. [61] report, the accumulation was initiated precisely at the time of cell growth inhibition under different conditions. This applied particularly with reduced nitrogen content; another species of microscopic filamentous fungi, *Mucor circinelloides*, rapidly increased its lipid accumulation.

Based on the results of their experiment, Prajapati et al. [62] assume that this method of collection could be an effective, sustainable, economic and viable tool for the collection of microalgae and for the production of biofuels. In their view, the investigated process not only overcomes the obstacles associated with the collection of microscopic algae but also improves its potential and suitability for generating bioenergy through anaerobic digestion. Muradov et al. [54] pointed out additional benefits of the synergism of fungal pellets with algae when the lipid content was increased through joint cultivation through the own production of these organisms without the need for any genetic modification or further treatment. As Wrede et al. [63] reported, the cultivation of algal cells and the species *Aspergillus fumigatus* showed additive and synergistic effects on biomass production, lipid yield and the efficiency of wastewater bioremediation. The composition of fungal algae pellets and fatty acids suggests that it is possible to adapt and optimize them by co-cultivating different algae and fungi without the need for genetic modification.

Among the various types of solid biomass fuels, pellets and briquettes are the most commonly used solid biofuels, where, depending on the application, the relevant standards specify that the diameter of pellet fuels should be less than or equal to 25 mm [40,64,65]. The species *A. fumigatus* showed up to 90% flocculation after the first 24 h of co-cultivation with no apparent differences in flocculation efficiency between freshwater and seawater, motile and non-motile species [63].

In the case of using only micellar cells, a rapid decrease in the concentration of ammonium ions took place. In the case of the supposed synergistic effect of the fungus and algae, however, there was an increase in the monitored ammonium ions. The reason is the disruption of the algal cell wall (proteins, polysaccharides) resulting from the production of organic acids (reduction in pH) produced by pellets of the *A. niger*. Complex amino acid chains break down into simpler ones, causing a release of ammonia ions. The synergism of the fungus and algae had an added value in the form of coagulation of *Chlorella* sp.

## 5. Conclusions

Although the original idea of this study was the decontamination of ammonium water, we achieved some interesting results during the experiment. The use of pellets of the filamentous fungus *A.niger* showed the potential for using this method in the process of collecting used microorganisms in wastewater treatment. As a consequence of adhesive forces, the algae culture *Chlorella* sp. was used on the surface of the pellets, which almost completely collects the used biomass from the solution, while the flocculation activity is approximately 70 to 80%.

In this study, an innovative mechanism for the collecting of SCO microorganisms for simplifying the harvesting and increasing biomass yield by means of a pelletized area was investigated. This technology uses the ability to pelletize microscopic filamentous fungi. The path of biocoagulation of algae in the presence of pellets also has, aside from the isolation of algae from an aqueous solution in the form of a coagulant (low costs), an added value in the created energy-efficient biomass (algae-fungus).

Chemical analyses also presented another interesting use of this method, which brings a potential ecological innovation in the area of biofuel production. Algae and microscopic filamentous fungi in the process of decontamination are able to remove inorganic as well as organic substances from the environment and are able to accumulate and be used as resource inputs for the benefit of the creation of their own biomass. The pellets do not have a positive charge, rather they are positive near the isoelectric point (very slightly). For this reason, pellets can be successful algae traps.

The added value, in addition to the reduced economic costs for the process of decontamination and biosorbent removal, is the use of the resulting biomass for energy conversion, or the energy of carriers through a series of conversion processes. These include thermochemical conversions (gasification, direct combustion and pyrolysis), biochemical conversions (anaerobic fermentation, anaerobic digestion and photobiological hydrogen production) and esterification of fatty acids to produce biodiesel. We thus obtain a process for using a biosorbent in a waste-free circulation system. From the water decontamination process, waste biomass has the potential to be used as a source for biogas stations. Biomass (algae and fungi) has a relatively high proportion of carbon and nitrogen content for the production of methane and hydrogen.

## Figures and Tables

**Table 1:** Parameters of used waters.

Water Matrix	Parameters							
	THC (mg.L^−1^)	COD (mg.L^−1^)	DSS_105 °C_ (mg.L^−1^)	DPI (mg.L^−1^)	pH	DDS_105°C_ (mg.L^−1^)	DA (mg.L^−1^)	BOD_5_ (mg.L^−1^)
Destiled water	<0.02	<0.02	<0.02	<0.02	6.80	<0.02	<0.02	<0.02
Model water	<0.02	<0.02	<0.02	<0.02	6.80	<0.02	9.00	<0.02
Wastewater	29.40	245	50.00	0.66	7.70	440.00	9.14	100

THC—Total Hydrocarbon Content, COD—Chemical Oxygen Demand, DSS—Determination of suspended solids, DPI—Determination of phenol index, pH—Determination of pH, DDS—Determination dissolved substances, DA—Determination of ammonium, BOD_5_—Determination of biochemical oxygen demand.

**Table 2:** Measurement results of the charge of particles.

Time of Biocoagulation (hours)	Specific Charge Density (meq/g)					
	Distilled Water + Algae Biomass (g/L)		Solution of Ammonium Ions 9 Mg/L + Algae Biomass (g/L)		Solution of Ammonium Ions 9 Mg/L + Algae Biomass (g/L) + 5 g/L Pellets	
* 0.5 g/L	5 g/L	0.5 g/L	5 g/L	0.5 g/L	5 g/L
24 h	0.05	0.02	0.25	0.32	0.39	0.13
48 h	0.09	0.03	0.20	0.38	0.33	0.13
72 h	0.05	0.04	0.21	0.39	0.18	0.16

* Concentration of algae biomass

**Table 3:** Measured values of selected biogenic elements after flocculation (72 h).

Biomass of *Chlorella* sp. (g/L) and *A. Niger* Pellets in Ammonium Ion Solution 9 mg/L	Time of Biocoagulation (Hours)	Content of Selected Elements			
Concentration of algae biomass (0.5 g/L)		C (%)	H (%)	N (%)	S (%)
	0	19.46	2.77	3.20	0.02
	72	20.83	2.04	4.38	0.1
Concentration of algae biomass (5 g/L)	0	23.85	3.07	3.58	0.04
	72	34.18	3.85	5.64	0.05

## Data Availability

Not applicable.

## References

[1] Megharaj M., Avudainayagam S., Naidu R. (2003). Toxicity of hexavalent chromium and its reduction by bacteria isolated from soil contaminated with tannery waste. Curr. Microbio..

[2] Dwivedi S. (2012). Bioremediation of heavy metal by algae: Current and future perspective. J. Adv. Lab. Res. Biol..

[3] Chisti Y. (2007). Biodiesel from microalgae. Biotechnol. Adv..

[4] Deshmukh S., Kumar R., Bala K. (2019). Microalgae biodiesel: A review on oil extraction, fatty acid composition, properties and effect on engine performance and emissions. Fuel Process. Technol..

[5] Clarens A.F., Resurreccion E.P., White M.A., Colosi L.M. (2010). Environmental life cycle comparison of algae to other bioenergy feedstocks. Environ. Sci. Technol..

[6] Zeraatkar A.K., Ahmadzadeh H., Talebi A.F., Moheimani N.R., McHenry M.P. (2016). Potential use of algae for heavy metal bioremediation, a critical review. J. Environ. Manag..

[7] Lal A., Banerjee S., Das D. (2021). Aspergillus sp. assisted bioflocculation of Chlorella MJ 11/11 for the production of biofuel from the algal-fungal co-pellet. Sep. Purif. Technol..

[8] Uduman N., Qi Z., Danquah M.K., Hoadley A.F.A. (2010). Marine microalgae flocculation and focused beam reflectance measurement. Chem. Eng. J..

[9] Rao P.H., Kumar R.R., Raghavan B.G., Subramanian V.V., Sivasubramanian V. (2011). Application of phycoremediation technology in the treatment of wastewater from a leather-processing chemical manufacturing facility. Water SA.

[10] Sengar R.M.S., Singh K.K., Singh S. (2011). Application of phycoremediation technology in the Treatment of sewage water to reduce pollution load. Indian J. Sci. Res..

[11] Bwapwa J.K., Jaiyeola A.T., Chetty R. (2017). Bioremediation of acid mine drainage using algae strains: A review. S Afr. J. Chem. Eng..

[12] Pragya N., Pandey K.K., Sahoo P.K. (2013). A review on harvesting, oil extraction and biofuels production technologies from microalgae. Renew. Sustain. Energy Rev..

[13] Amer L., Adhikari B., Pellegrino J. (2011). Technoeconomic analysis of five microalgae-to-biofuels processes of varying complexity. Biosour. Technol..

[14] Branyikova I., Prochazkova G., Potocar T., Jezkova Z., Branyik T. (2018). Harvesting of microalgae by flocculation. Fermentation.

[15] Kwon H., Lu M., Lee E.Y., Lee J. (2014). Harvesting of microalgae using flocculation combined with dissolved air flotation. Biotechnol. Bioprocess Eng..

[16] Mo W., Soh L., Werber J.R., Elimelech M., Zimmerman J.B. (2015). Application of membrane dewatering for algal biofuel. Algal Res..

[17] Hapońska H., Clavero E., Salvado J., Farriol X., Torras C. (2018). Pilot scale dewatering of Chlorella sorokiniana and Dunaliella tertiolecta by sedimentation followed by dynamic filtration. Algal Res..

[18] Feiyan Z., Junmu X., Wei D., Na C., Xuya Y., Jun-Wei X., Tao L., Peng Z. (2019). An effective method for harvesting of microalga: Coculture-induced self-flocculation. J. Taiwan Inst. Chem. Eng..

[19] De Hoog G.S., Guarro J., Gené J., Ahmed S.A., Al-Hatmi A.M.S., Figueras M.J., Vitale R.G. (2020). Atlas of Clinical Fungi the Ultimate Benchtool for Diagnostics.

[20] Šimonovicová A., Vojtková H., Nosalj S., Piecková E., Švehláková H., Kraková L., Drahovská H., Stalmachová B., Kučová K., Pangallo D. (2021). Aspergillus niger Environmental Isolates and Their Specific Diversity Through Metabolite Profiling. Front. Microbiol..

[21] Nosalj S., Šimonovičová A., Vojtková H. (2021). Enzyme production by soilborne fungal strains of Aspergillus niger isolated from different localities affected by mining. IOP Conference Series: Earth and Environmental Science.

[22] Šimonovicová A., Takáčová A., Šimkovic I., Nosalj S. (2021). Experimental treatment of hazardous ash waste by microbial consortium Aspergillus niger and *Chlorella* sp.: Decrease of the Ni content and identification of adsorption sites by Fourier-Transform Infrared spectroscopy. Front. Microbiol..

[23] Nosalj S., Jesenák K., Šimonovičová A., Hudec P. (2021). Impact of inorganic powder additives on the size and morphology of pellets of the microscopic filamentous fungus Aspergillus niger. Nova Biotechnol. Chim..

[24] Zhou W., Cheng Y., Li Y., Wan Y., Liu Y., Lin X., Ruan R. (2012). Novel fungal pelletization-assisted technology for algae harvesting and wastewater treatment. Appl. Biochem. Biotechnol..

[25] Alrubaie G., Al-Shammari R.H.H. (2018). Microalgae Chlorella vulgaris harvesting via co-pelletization with filamentous fungus. Baghdad. Sci. J..

[26] Li S., Hu T., Xu Y., Wang J., Chu R., Yin Z., Mo F., Zhu L. (2020). A review on flocculation as an efficient method to harvest energy microalgae: Mechanisms, performances, influencing factors and perspectives. Renew. Sustain. Energy. Rev..

[27] Kamyab H., Lee C.T., Chelliapan S., Khademi T., Talaiekhozani A., Rezania S. (2019). Role of Microalgal Biotechnology in Environmental Sustainability-A Mini Review. Chem. Eng. Trans..

[28] Kamyab H., Tin L.C.H., Din M.F.M., Ponraj M., Mohamad S.E., Sohrabi M. (2014). Effects of nitrogen source on enhancing growth conditions of green algae to produce higher lipid. Desalination Water Treat..

[29] Šimonovičová A., Kraková L., Pauditšová E., Pangallo D. (2019). Occurrence and diversity of cultivable autochthonous microscopic fungi in substrates of old environmental loads from mining activities in Slovakia. Ecotoxicol. Environ. Saf..

[30] Domsch K.H., Gams W., Anderson T. (2007). Compendium of Soil Fungi.

[31] Klich M.A. (2002). Biogeography of Aspergillus species in soil and litter. Mycologia.

[32] Šutý Š., Lužáková V. Some aspects of ground calcium carbonate deposition onto pulp fibres. Proceedings of the INPAP′97 Kierunki Rozwoju Technologii I Konstrukcji Maszyn W Papiernictwie.

[33] Li Y., Xu Y., Liu L., Li P., Yan Y., Chen T., Zheng T., Wang H. (2017). Flocculation mechanism of Aspergillus niger on harvesting of *Chlorella* vulgaris biomass. Algal Res..

[34] Šutý Š., Lužáková V. (1998). Role of surface charge in deposition of filler particles onto pulp fibers. Colloids Surfaces. Physicochem. Eng. Asp..

[35] Lužáková V., Marcinčinová T., Šutý Š. Possibilities of using particle surface charge measurement in pulp and paper production. Proceedings of the 53rd Congress of Chemical Societies.

[36] Takáčová A., Smolinská M., Ryba J., Mackuľak T., Jokrlová J., Hronec P., Čík G. (2014). Biodegradation of benzo[a]pyrene through the use of algae. Cent. Eur. J. Chem..

[37] French C.S., Pirson A. (1960). The chlorophylls in vivo and in vitro. Die CO_2_-Assimilation/The Assimilation of Carbon Dioxide.

[38] Slomkowski S., Alemán J.V., Gilbert R.G., Hess M., Horie K., Jones R.G., Kubisa P., Meisel I., Mormann W., Penczek S. (2011). Terminology of polymers and polymerization processes in dispersed systems. Pure Appl. Chem..

[39] Guggenheim A., Haller R. (1972). Purification and properties of an α-(1 → 3) glucanohydrolase from Trichoderma harzianum. J. Dent. Res..

[40] Zhang J., Hu B. (2012). A novel method to harvest microalgae via co-culture of filamentous fungi to form cell pellets. Biosour. Technol..

[41] Metz A., Kossen N.W.F. (1977). The growth of molds in the form of pellets–a literature review. Biotechnol. Bioeng..

[42] Braun S., Vechtlifshitz S.E. (1991). Mycelial morphology and metabolite production. Trends Biotechnol..

[43] Grimm L.H., Kelly S., Hengstler J., Gobel A., Krull R., Hempel D.C. (2004). Kinetic studies on the aggregation of Aspergillus niger conidia. Biotechnol. Bioeng..

[44] Zmak P.M., Podgornik A., Podgornik H., Koloini T. (2006). Impact of pellet size on growth and lignin peroxidase activity of Phanerochaete chrysosporium. World J. Microbiol. Biotechnol..

[45] Krull R., Cordes C., Horn H., Kampen I., Kwade A., Neu T.R., Nortemann B. (2010). Morphology of filamentous fungi: Linking cellular Biology to process engineering using Aspergillus niger. Biosys. Eng..

[46] Gultom S.O., Hu B. (2013). Review of microalgae harvesting via co-pelletization with filamentous fungus. Energies.

[47] Pahl S.L., Lee A.K., Kalaitzidis T., Ashman P.J., Sathe S., Lewis D.M. (2013). Harvesting, thickening and dewatering microalgae biomass. Algal Biofuel Energy.

[48] Vandamme A., Foubert I., Muylaert K. (2013). Flocculation as a low-cost method for harvesting microalgae for bulk biomass production. Trends Biotechnol..

[49] Wan C., Alam M.A., Zhao X.Q., Zhang X.Y., Guo S.L., Ho S.H., Chang J.S., Bai F.W. (2015). Current progress and future prospect of microalgal biomass harvest using various flocculation technologies. Biosour. Technol..

[50] Matter I.A., Bui V.K.H., Jung M., Seo J.Y., Kim Y., Lee Y., Oh Y. (2019). Harvesting of microalgae S. obliquus using chitosan-alginate dual flocculation system. App. Sci..

[51] Bratby J. (2006). Coagulation and Flocculation in Water and Wastewater Treatment.

[52] Salim S., Kosterink N.R., Tchetkoua Wacka N.D., Vermue M.H., Wijffels R.H. (2014). Mechanism behind autoflocculation of unicellular green microalgae Ettlia texensis. J. Biotechnol..

[53] Sanyano N., Chetpattananondh P., Chongkhong S. (2013). Coagulation–flocculation of marine Chlorella sp. for biodiesel production. Biosour. Technol..

[54] Muradov N., Taha M., Miranda A.F., Wrede D., Kadali K., Gujar A., Stevenson T., Ball A.S., Mouradov A. (2015). Fungal-assisted algal flocculation: Application in wastewater treatment and biofuel production. Biotechnol. Biofuels.

[55] McKendry P. (2002). Energy production from biomass (part 3): Gasification technologies. Biosour. Technol..

[56] Melis A. (2002). Green alga hydrogen production: Progress, challenges and 3 prospects. Int. J. Hydrogen Energy.

[57] Miao X.L., Wu Q.Y. (2004). High yield bio-oil production from fast pyrolysis by metabolic controlling of Chlorella protothecoides. J. Biotechnol..

[58] Pittman J.K., Dean A.P., Osundeko O. (2011). The potential of sustainable algal biofuel production using wastewater resources. Biosour. Technol..

[59] Miao X.L., Wu Q.Y. (2006). High quality biodiesel production from a microalga Chlorella protothecoides by heterotrophic growth in fermenters. Biosour. Technol..

[60] Liang N.Y., Sarkany N., Cui Y. (2009). Biomass and lipid productivities of Chlorella vulgaris under autotrophic, heterotrophic and mixotrophic growth conditions. Biotechnol. Lett..

[61] Xia C., Zhang J., Zhang W., Hu B. (2011). A new cultivation method for microbial oil production: Cell pelletization and lipid accumulation by Mucor circinelloides. Biotechnol. Biofuels.

[62] Prajapati S.K., Malik A., Vija V.K. (2014). Comparative evaluation of biomass production and bioenergy generation potential of Chlorella spp. through anaerobic digestion. Appl. Energy.

[63] Wrede D., Taha M., Miranda A.F., Kadali K., Stevenson T., Ball A.S., Mouradov A. (2014). Co-Cultivation of fungal and microalgal cells as an efficient system for harvesting microalgal cells, lipid production and wastewater treatment. PLoS ONE.

[64] Pradhan P., Mahajani S.M., Arora A. (2018). Production and utilization of fuel pellets from biomass: A review. Fuel Process. Technol..

[65] Cui J., Wang G., Liu W., Ke P., Tian Q., Li X., Tian Y. (2021). Synthesis BiVO4 modified by CuO supported onto bentonite for molecular oxygen photocatalytic oxidative desulfurization of fuel under visible light. Fuel.

